# Hippo-YAP/MCP-1 mediated tubular maladaptive repair promote inflammation in renal failed recovery after ischemic AKI

**DOI:** 10.1038/s41419-021-04041-8

**Published:** 2021-07-30

**Authors:** Zhihuang Zheng, Chuanlei Li, Guangze Shao, Jinqing Li, Kexin Xu, Zhonghua Zhao, Zhigang Zhang, Jun Liu, Huijuan Wu

**Affiliations:** 1 Department of Nephrology, Shanghai General Hospital, Shanghai Jiaotong University School of Medicine, Shanghai, China; 2 Department of Pathology, School of Basic Medical Sciences, Fudan University, Shanghai, China

**Keywords:** Chronic inflammation, Acute kidney injury

## Abstract

Acute kidney injury (AKI) is associated with significant morbidity and its chronic inflammation contributes to subsequent chronic kidney disease (CKD) development. Yes-associated protein (YAP), the major transcriptional coactivator of the Hippo pathway, has been shown associated with chronic inflammation, but its role and mechanism in AKI-CKD transition remain unclear. Here we aimed to investigate the role of YAP in AKI-induced chronic inflammation. Renal ischemia/reperfusion (I/R) was used to induce a mouse model of AKI-CKD transition. We used verteporfin (VP), a pharmacological inhibitor of YAP, to treat post-IRI mice for a period, and evaluated the influence of YAP inhibition on long-term outcomes of AKI. In our results, severe IRI led to maladaptive tubular repair, macrophages infiltration, and progressive fibrosis. Following AKI, the Hippo pathway was found significantly altered with YAP persistent activation. Besides, tubular YAP activation was associated with the maladaptive repair, also correlated with interstitial macrophage infiltration. Monocyte chemoattractant protein 1 (MCP-1) was found notably upregulated with YAP activation. Of note, pharmacological inhibition of YAP in vivo attenuated renal inflammation, including macrophage infiltration and MCP-1 overexpression. Consistently, in vitro oxygen-glucose deprivation and reoxygenation (OGD/R) induced YAP activation and MCP-1 overproduction whereas these could be inhibited by VP. In addition, we modulated YAP activity by RNA interference, which further confirmed YAP activation enhances MCP-1 expression. Together, we concluded tubular YAP activation with maladaptive repair exacerbates renal inflammation probably via promoting MCP-1 production, which contributes to AKI-CKD transition.

## Introduction

Acute kidney injury (AKI) and chronic kidney disease (CKD) have a substantial impact on morbidity and mortality and represent an important challenge in global public health [1]. Importantly, incomplete recovery from AKI, also called maladaptive repair, can lead to long-term functional deficits and progressive pathological transition to CKD [2–4]. Several studies demonstrated maladaptive tubular repair contributes to cytokine secretion, inflammation, production of extracellular matrix, and progressive renal interstitial fibrosis [4–6]. Of note, the maladaptive tubular repair was reported to crosstalk with immune cells, which further results in intrarenal persistent cellular infiltration and tubulointerstitial fibrosis [4, 7–10]. However, the cellular or molecular pathway mediating this crosstalk is poorly understood.

The mammalian Hippo pathway is a serine/threonine kinase signaling with a three-step cascade composed of mammalian sterile 20-like kinase 1/2 (MST1/2), large tumor suppressor 1/2 (LATS1/2), and Yes-associated protein (YAP) [11]. Hippo pathway is implicated in the regulation of cell proliferation, differentiation, and death by phosphorylating and inactivating downstream effector YAP [12, 13]. YAP serves as an important transcriptional coactivator, which can enter the nucleus and bind with TEA domains (TEAD) being a complex to regulate genes expressions. Although recent evidence strongly suggests the Hippo pathway plays a critical role in regulating inflammation in other tissues [14–20], its role in AKI inflammation remains unknown. In addition, Chen et al. found YAP activation after AKI mediates renal epithelial cell repair and regeneration [21]. Given that, we hypothesized that the Hippo pathway might play important roles in tubular maladaptive repair and inflammation following AKI.

To investigate our hypothesis, the AKI-CKD model was induced by ischemia/reperfusion injury and the correlation between inflammatory infiltration and Hippo pathway in post-AKI kidney tissue was also assessed. Next, we used the pharmacological inhibitor verteporfin (VP) to treat post-IRI mice for a period, and evaluated the effects of YAP inhibition on tubular maladaptive repair and inflammation following AKI. Furthermore, in combination with Gene Expression Omnibus (GEO) analysis and YAP modulation in vitro, we demonstrated the molecular mechanism of Hippo-YAP influencing inflammation in AKI-CKD transition.

## Materials and methods

### Mice

To prepare animal models, 8-week-old male C57BL/6 mice weighing 23–25 g were purchased (SLAC Laboratory Animal Co., Shanghai Laboratory Animal Center). The mice were housed in a specific pathogen-free environment at the Animal Center of the Shanghai General Hospital at the optimal temperature with a 12 h light/12 h dark cycle. All animal experiments were performed in strict accordance with the guidelines of the National Institutes of Health Guide for the Care and Use of Laboratory Animals and were approved by the Ethics Committee of Shanghai General Hospital, Shanghai Jiaotong University School of Medicine (Shanghai, China).

### In vivo renal ischemia/reperfusion model

The ischemia/reperfusion (I/R)-induced AKI mouse model was established as previously described [22]. Briefly, the mice were randomly assigned and anaesthetized intraperitoneally with pentobarbital sodium (6 mg/100 g). The renal pedicles were then exposed by a midline incision. I/R injury were induced after right uninephrectomy by clipping the pedicles of the remaining left kidney for 20 m with a nontraumatic aneurysm clip (Rivard Life Science Co. Ltd., Shenzhen, China). The clamps were released and reperfusion was confirmed visually. Sham control mice underwent the same operation without renal pedicle clamping. During induction of ischemic AKI, the body temperatures of the mice were controlled at ~37 °C. Pre-warmed saline solution (0.5 ml; 37 °C) was then administered intraperitoneally before the abdomen was closed. Preemptive analgesia with buprenorphine (0.2 mg/100 g) was applied subcutaneously to every mouse. The mice were sacrificed on days 14 and 28 after renal surgery (*n* = 6/group). Blood samples and kidney tissues were collected rapidly for further analysis. The kidneys were divided into three sections. One third of the kidney was snap-frozen in liquid nitrogen for western blot, one third was immersed in 4% PBS-buffered formalin for histology and immunohistochemistry, and the rest was snap-frozen in liquid nitrogen for qRT-PCR.

### Cell culture and oxygen-glucose deprivation and reoxygenation injury

Human kidney 2 cells (HK-2; FuHeng Cell Center, Shanghai, China), a proximal tubular cell line derived from normal kidneys, and murine renal tubular epithelial cell line (TEC), were cultured in Dulbecco’s modified Eagle’s medium/nutrient mixture F-12 (HyClone Laboratories, Logan, UT, USA) with 10% fetal bovine serum (Thermo Fisher Scientific, Waltham, MA, USA), 100 μg/mL streptomycin (Thermo Fisher Scientific), and 100 U/mL penicillin (Thermo Fisher Scientific). And additionally, EGF with a final concentration of 2.5 ng/ml (Sigma-Aldrich, St. Louis, USA) was needed for culturing of HK-2. To simulate ischemia/reperfusion injury in vitro, we used a model of oxygen-glucose deprivation followed by reoxygenation (OGD/R). OGD/R cell model was established as previously described with slight modification [23, 24]. HK-2 cells or TEC cells were cultured in glucose-free DMEM (GIBCO) and exposed to a hypoxia incubator chamber (STEMCELL Technologies, Vancouver, BC, Canada) with an anoxic mixture gas (95% N2 and 5% CO2) for 12 h at 37 °C. Then cells were returned to normal medium and placed in a normoxic chamber (37 °C, 5% CO2) for 2 h of reoxygenation.

### RNA interference in vitro

The LATS1 shRNA and YAP shRNA were purchased from Gene Pharma (Shanghai, China). HK2 cells were grown in DMEM/F12 medium supplemented with 10% FBS in coning 24-well dishes, after which the HK2 cells were transfected with the LATS1 or YAP shRNA. Forty-eight hours after transfection using the TransIT-X2 Dynamic Delivery System (MIRUS, Madison, WI, USA), the cells were made quiescent in FBS-free medium for 6 h and then transferred to DMEM/F12 medium with 10% FBS. The cells were lysed in radioimmunoprecipitation assay (RIPA; Sheng Gong Biotech, Shanghai, China) buffer for western blotting analysis.

### Pharmacological inhibitor

To inhibiting YAP activity, we used 25 mg/kg, 10 µM of VP in vivo and in vitro, respectively. In vivo, mice were intraperitoneally administered with VP on the 3rd day after surgery and every 72 h for 25 days. In vitro, cells were pretreated by VP for 6 h. In addition, we also used 20 µM of lysophosphatidic acid (LPA) which is an agonist of YAP for 12 h in vitro and vehicle as control.

### Serum measurements

Eighty milliliters of facial blood were collected on days 1, 7, 14, and 28 as indicated time points, respectively. Creatinine (Cr) and blood urea nitrogen (BUN) chemical kits and MCP-1 ELISA kit were purchased from Jiancheng Bioengineering Institute of Nanjing (Nanjing, China). Experiments were performed according to the manufacturer’s protocol and repeated three times for each indicator.

### Western blotting

Kidney tissues were harvested from mice with different groups and were lysed with RIPA buffer (Sigma-Aldrich, St. Louis, MO) supplemented with multiple protease inhibitors (Roche, Basel, Switzerland). Fifty-microgram protein samples were separated by 12% SDS-PAGE. After semidry transfer, nonspecific binding sites of the nitrocellulose membrane were blocked with 5% nonfat milk in Tris-buffered saline containing 0.1% Tween. After that, the membrane was incubated with the following primary antibodies: monoclonal rabbit anti-YAP (Cat# 14074 S, CST, USA), monoclonal rabbit anti-phospho-YAP (ser127) (Cat# 13008 S; CST, USA), monoclonal rabbit anti-LATS1 (Cat# 3477 S, CST, USA), monoclonal rabbit anti-TEAD (Cat# 10511 S, CST, USA), monoclonal rabbit anti-α-SMA (Cat# 19245 S, CST, USA), monoclonal rabbit anti-MCP-1 (Cat# ab25124 and ab214819, Abcam, UK). Next, immunoblots were processed with appropriate secondary antibodies for 1 h at room temperature. Blots were analysed with the enhanced chemiluminescence (ECL) system and captured on autoradiographic films. These films were scanned, and density analysis of the bands was performed with Labworks 4.6 Analysis System (Labworks, Inc., USA). Glyceraldehyde 3-phosphate dehydrogenase (GADPH) and β-Actin were blotted on the same membrane as loading controls.

### Histological analysis

Formalin-fixed, paraffin-embedded, 2-mm-thick kidney sections were stained with haematoxylin and eosin (H&E) and Sirius Red using standard protocols. The severity of tubulointerstitial fibrosis was assessed by a renal pathologist blinded to the mice groups. The findings were graded from 0 to 3 according to the distribution of lesions: 0, no lesion; 1, less than 20%; 2, 20–50%; 3, more than 50%. Scoring was performed in a blinded fashion in ten consecutive fields at a magnification of 400× per section. All tests were repeated three times.

### Immunohistochemistry and immunofluorescence

The kidneys were removed and fixed in 4% paraformaldehyde, embedded in paraffin, and cut into 2-μm-thick sections. Immunohistochemistry was performed to assess renal tubular injury and fibrosis. Immunofluorescence was performed to assess renal inflammatory infiltration of macrophages. Immunohistochemistry and immunofluorescence staining of the kidney was performed on paraffin sections as previously described [21, 25]. Co-staining of immunofluorescence was performed using the Opal 4-color manual immunohistochemistry kit (Perkin Elmer, USA) according to the manufacturer’s instructions. Sections were stained with the following antibodies: monoclonal rabbit anti-YAP (Cat# 14074 S, CST, USA), monoclonal mouse anti-E-cadherin (Cat#14472, CST, USA), monoclonal rabbit anti-F4/80 (Cat# 70076 T, CST, USA), and monoclonal rabbit anti-MCP-1 (Cat# ab25124, Abcam, UK). Positive cells were counted in the outer medulla on five nonoverlapping view fields at 400× magnification and mean cell numbers were taken for analysis.

### Quantitative real-time PCR

qRT-PCR was performed as described previously [26]. Total RNA from snap-frozen kidneys or HK-2 cells was isolated using an RNeasy RNA isolation kit (Qiagen, Australia). The concentration and quality of RNA were measured by a NanoDrop-1000 spectrophotometer (Thermo Fisher Scientific, USA). Next, 2 µg of RNA was used for cDNA transcription (Applied Biosystems, USA). The quantitative analysis for target mRNA expression was performed with qRT-PCR using the relative standard curve method. SYBR green analysis was conducted using an Applied Biosystems 7500 Sequence Detector (Applied Biosystems, USA). The expression levels were normalized to *β-Act*. Primer sequences are provided in Supplementary Table S1.

### Statistical analyses

Statistical analyses were performed using GraphPad 5.0 (GraphPad Software, La Jolla, CA) software. Unpaired *t*-test was used in the case of two groups, as stated in figure legends. In case of more than two groups, data were analyzed by one-way ANOVA using Tukey’s post hoc test. Data were presented as mean ± SEM. *P* values < 0.05 were considered to represent statistically significant differences.

## Results

### Severe ischemic AKI induced tubular maladaptive repair with macrophages infiltration and tubulointerstitial fibrosis

**Fig. 1:**
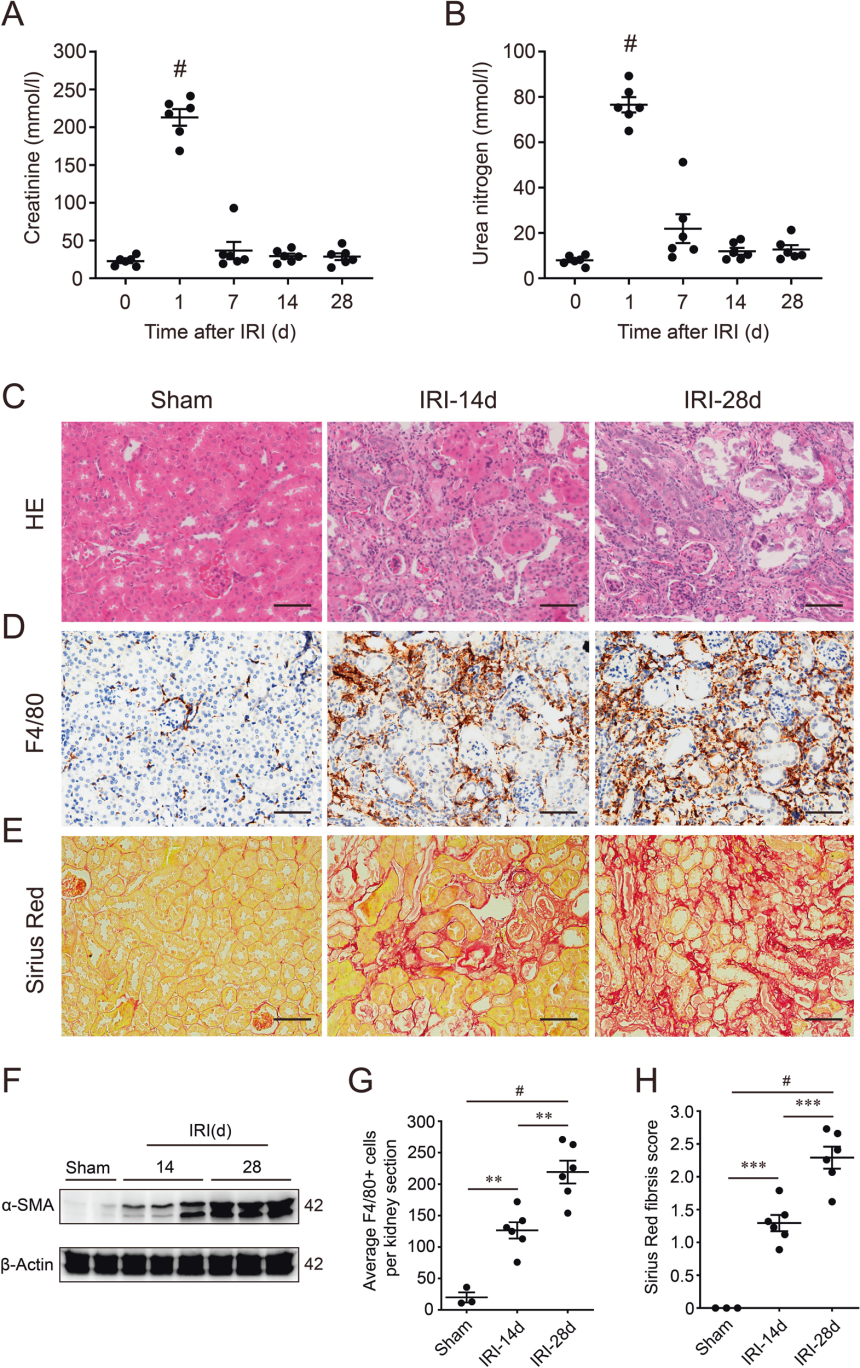
Severe ischemia/reperfusion-induced acute kidney injury (AKI) led to renal maladaptive repair with chronic inflammation and fibrosis. **A** Serum creatinine and **B** urea nitrogen levels followed IRI (*n* = 6 mice). The serum creatinine and urea nitrogen levels on day 0 represent normal baseline measured before surgery. #*p* < 0.001 defined as significant to baseline. **C** Representative images of HE staining on Sham, IRI-14d, and IRI-28d kidneys. **D** Representative images of F4/80 immunohistochemistry staining on Sham, IRI-14d, and IRI-28d kidneys. **E** Representative images of Sirius Red staining on Sham, IRI-14d, and IRI-28d kidneys. **F** Renal expression of α-SMA in Sham, IRI-14d, and IRI-28d mice. Shown are representative blots from at least three separate experiments with similar results. **G** Quantification of F4/80-positive cells in kidney (counted). (*n* = 3 for Sham, and *n* = 6, 6 for IRI-14d and IRI-28d, respectively). **H** Semi-quantification of Sirius Red staining. (*n* = 3 for Sham, and *n* = 6, 6 for IRI-14d and IRI-28d, respectively). Scale bar = 50 µm in all images of **C**, **D**, and **E**. All values are means ± SEM. **p < 0.01 and ^#^*p* < 0.001 are defined as significant.

A unilateral IRI mouse model after uninephrectomy was used to assess the renal long-term outcome of AKI. The serum levels of serum Cr and BUN was significantly increased in 24 h after IRI, compared to the normal baseline (Fig. 1A, B). However, the serum level of Cr and BUN was declined subsequently from the 2nd day (Supplementary Fig. S1A, B), and eventually equivalent to baseline after 7 days (Fig. 1A, B). Although renal function recovered basically, renal histology showed excessive maladaptive repair until 28 days after IRI. As shown in Fig. 1C, post-AKI mice showed abnormal tubules with an accumulation of immune cells and progressive fibrosis. Maladaptive tubular repair represented atrophic, flattening epitheliums, loss of brush border, luminal dilatation (Fig. 1C). In the first 3 days after IRI, we found the number of F4/80-positive macrophages, and expression of α-smooth muscle actin (α-SMA), a representative myofibroblasts marker, was starting to increase (Supplementary Fig. S1C). Furthermore, 14 or 28 days after IRI, the number of infiltrating F4/80-positive macrophages increased more significantly (Fig. 1D, G). And Sirius Red staining revealed a significant exacerbation of interstitial fibrosis at the 14th or 28th day after IRI (Fig. 1E, H). Consistently, western blot also showed the renal cortex expression of α-SMA increased in 14 or 28 days post-AKI mice, compared to a sham group (Fig. 1F). Hence, severe IRI could cause renal maladaptive repair with progressive inflammation and fibrosis over time.

### Hippo-YAP pathway was activated persistently in response to renal maladaptation following AKI

**Fig. 2:**
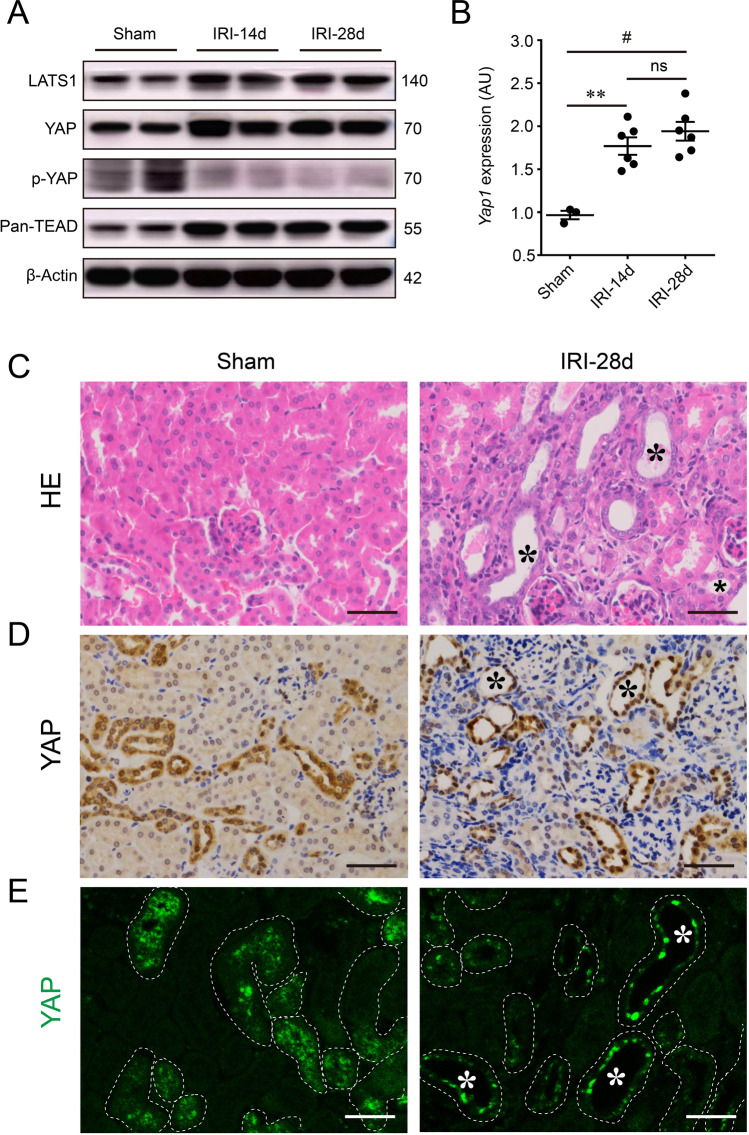
Renal persistent activation Hippo-YAP pathway in response to AKI-induced maladaptation. **A** Renal expressions of LATS1, YAP, p-YAP(ser127), and TEAD in Sham, IRI-14d, and IRI-28d mice. Shown are representative blots from at least three separate experiments with similar results. **B** Renal mRNA level of *Yap1*. AU arbitrary units. (*n* = 3 for Sham and *n* = 6, 6 for IRI-14d and IRI-28d, respectively). **C** Representative images of renal cortex stained with HE. Asterisk represents pathological changes of tubular maladaptive repair in Sham and IRI-28d kidneys. **D** Representative images of immunohistochemistry and **E** immunofluorescent staining for YAP in Sham and IRI-28d kidneys. Asterisk represents the renal maladaptive-repaired tubules as well. Scale bar = 50 µm in all images of **C**, **D**, and **E**. All values are means ± SEM. **p < 0.01 and ^#^*p* < 0.001 are defined as significant. ns not significant.

To investigate the change of the Hippo pathway in response to AKI, we examined the core protein members of Hippo in kidneys following IRI. Western blot showed increased expression of LATS1, YAP, and TEAD, but decreased phosphorylation of YAP on the site of ser127 in 14 or 28 days post-IRI kidneys, compared with the sham mice, indicating YAP translocation from cytoplasm to nucleus and the activation of Hippo pathway after IRI (Fig. 2A). Furthermore, in post-AKI mice, renal *Yap1* mRNA expression also significantly increased after 14 and 28 days, compared with the sham group (Fig. 2B). Remarkably, immunohistochemistry and immunofluorescence staining showed that YAP distribution in the tubular epithelial nucleus was dramatically increased by the 28th day after IRI, indicating persistent activation and nuclear translocation of YAP (Fig. 2D, E). In combination with renal HE staining, tubular YAP activation was mainly observed in tubular epthelial cells with maladaptive repair (Fig. 2C, D). These tubules failed to recover normal structure become atrophic and surrounded by immune cells and fibrosis (Fig. 2C–E), implying tubular YAP activation associated with tubular maladaptive repair after IRI-induced AKI.

### Tubular YAP activation was associated with macrophages infiltration and MCP-1 upregulation following AKI

**Fig. 3:**
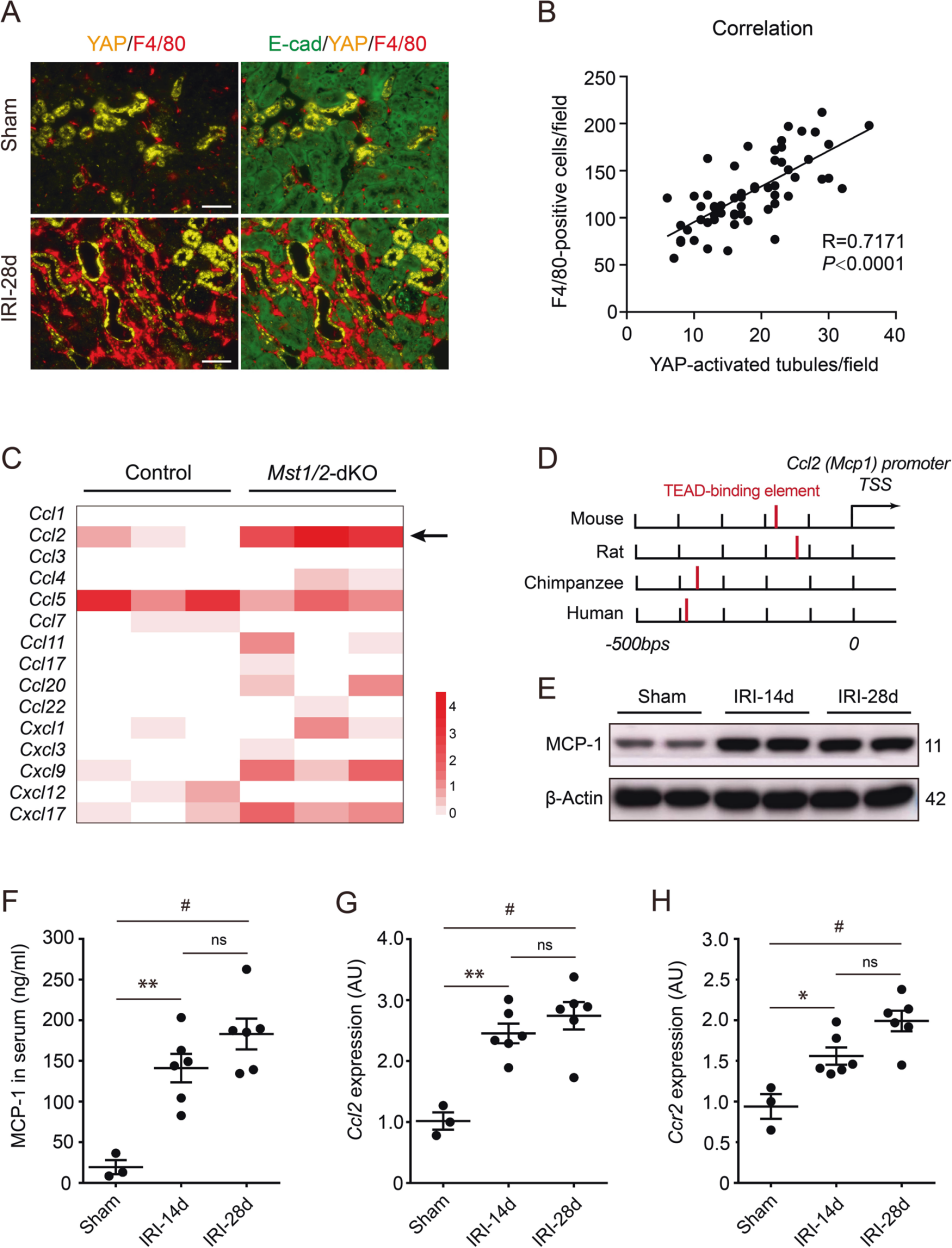
Tubular epithelial YAP activation associated with macrophage infiltration and renal MCP-1 upregulation. **A** Representative image of double immunofluorescent staining with YAP and F4/80 in Sham and IRI-28d kidneys. **B** YAP-activated tubules and F4/80-positive infiltrates were calculated from 55 randomly captured images at IRI-28d kidneys. Correlation between YAP-activated tubules and F4/80-positive infiltrates was analyzed (*n* = 55). Pearson correlation *R* value and *P* value are indicated. **C** Gene expression analysis for chemokine members including CCL and CXCL family in *Mst1/2* knockout mice. Gene Expression Omnibus (GEO) datasets supply the RNA-sequencing data of GES95463. **D** Regulatory elements of the genes encoding MCP-1 from various mammals were reported by Kim et al.[20]. The TEAD-binding element (red bar) is located 100–400 bp upstream of the transcription start site of MCP-1. TSS transcription start site. **E** Representative western blot analysis of MCP-1 protein expression in kidney lysates from mice 14 and 28 days after IRI (*n* = 6 mice per group). Sham-operated mice was used as a control (*n* = 3 mice). **F** ELISA analysis of MCP-1 levels in serum. **G**, **H** Quantitative polymerase chain reaction analysis of MCP-1 (*Ccl2*) and CCR2 (*Ccr2*) mRNA expression in kidneys. AU arbitrary units. Scale bar = 50 µm in all images of **A**. All values are means ± SEM. *p < 0.05, **p < 0.01, and ^#^*p* < 0.001 defined as significant. ns not significant.

Continuing studies are focusing on the role of Hippo in inflammation [18, 27]. Thus we put attention to the relationship between tubular YAP activation and renal inflammatory infiltration. Interestingly, double immunofluorescence with YAP and F4/80 showed that excessive macrophages were infiltrated adjacent to YAP-activated tubules (Fig. 3A). Pearson analysis showed infiltrating macrophages correlated to YAP-activated tubules (*R* = 0.7171; *P* < 0.0001), which indicates tubular YAP activation associated with macrophage recruitment (Fig. 3B). Next, we found a chemokine gene *Ccl2* encoding MCP-1, which was markedly enhanced in RNA-sequencing data from *Mst1/2* knockout mice (GES95463) (Fig. 3C). MST1/2 is an upstream regulator of LATS1/2, which controls LATS1/2 and further affects YAP activity [13, 14]. Notably, the binding element of TEAD, the YAP-downstream molecule, was reported to locate on 100–400 bp upstream of the transcription start site of *Ccl2* [20] (Fig. 3D). Thus, we examined renal MCP-1 after AKI. Western blot showed renal MCP-1 overexpressed following IRI-induced AKI (Fig. 3E). Serum MCP-1 also showed a notable increase in post-AKI mice, compared to sham mice (Fig. 3F). Similarly, Renal *Ccl2* and *Ccr2* (chemokine C-C motif receptor 2) were markedly increased in post-AKI mice versus sham groups (Fig. 3G, H). Hence, MCP-1 probably mediates YAP regulating renal macrophage infiltration after AKI.

### Inhibition of YAP in vivo attenuated renal macrophages infiltration and fibrosis after AKI

**Fig. 4:**
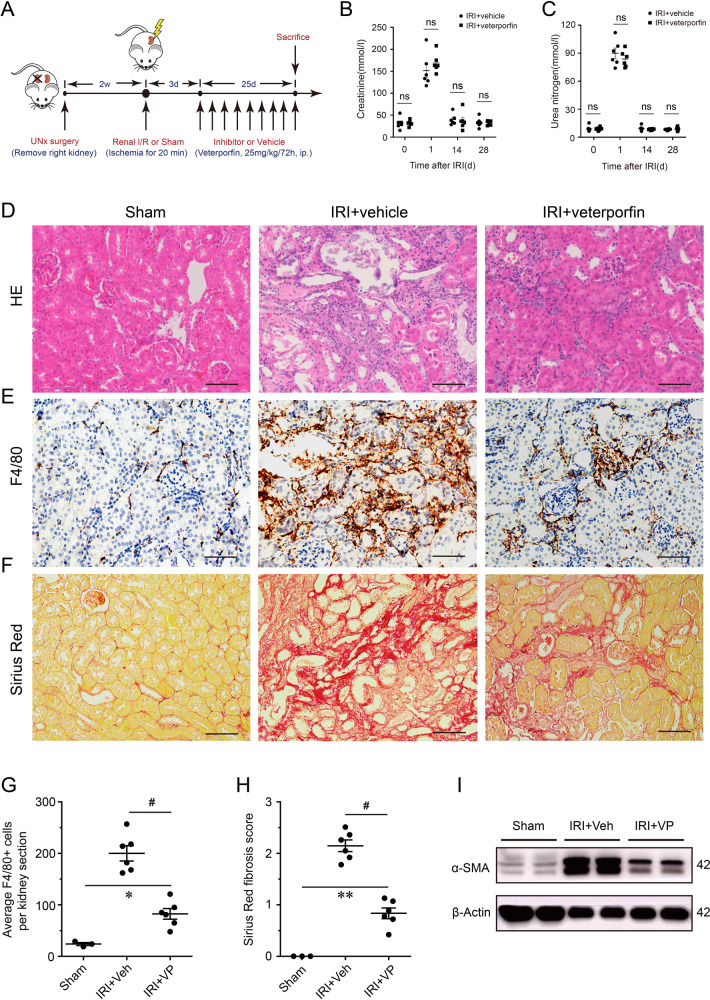
The impact of pharmacological inhibition of YAP on renal inflammatory infiltration and fibrosis after AKI. **A** Experimental design of YAP inhibition in ischemic AKI mice using verteporfin (25 mg/kg/72 h, i.p.). **B** The effect of verteporfin on serum creatinine and **C** urea nitrogen followed IRI (*n* = 6 mice). **D** Representative images of renal cortex stained with HE. **E** Representative images of immunohistochemistry staining for F4/80 in verteporfin treated mice and vehicle groups with AKI. **F** Representative images of renal Sirius Red staining. **G** Quantification of F4/80-positive cells in kidney (*n* = 3 for sham and *n* = 6, 6 for IRI + Veh and IRI + VP, respectively). **H** Semi-quantification of Sirius Red staining (*n* = 3 for sham and *n* = 6, 6 for IRI + Veh and IRI + VP, respectively). **I** Renal expression of α-SMA in Sham, IRI + Veh, and IRI + VP mice. Shown are representative blots from at least three separate experiments with similar results. Scale bar = 50 µm in all images of **D**, **E**, and **F**. All values are means ± SEM. *p < 0.05, **p < 0.01, and ^#^*p* < 0.001 defined as significant. ns not significant.

To further investigate the YAP function in renal inflammation, we used VP, a pharmacological inhibitor of YAP, to treat AKI mice and evaluate its long-term outcomes (Fig. 4A). The serum levels of Cr and BUN in VP-treated mice were similar to those in the vehicle group, which both underwent renal IRI (Fig. 4B, C). However, the renal histopathology revealed that the degree of maladaptive tubular repair and fibrosis was significantly reduced in VP-treated mice versus the vehicle group (Fig. 4D). Besides, immunochemistry staining for F4/80 showed the number of infiltrating macrophages was decreased in VP-treated mice compared to the vehicle group (Fig. 4E, G). Sirius Red staining also showed that VP administration significantly attenuated the interstitial fibrosis after AKI (Fig. 4F, H). Similarly, the expression of renal α-SMA has a notable reduction in VP-treated mice, compared with the vehicle group (Fig. 4I). Inhibiting YAP activity can attenuate renal macrophages infiltration and subsequent fibrosis after AKI, which suggests YAP activation is critical to renal AKI inflammation.

### In vivo increase of renal MCP-1 after ischemic AKI was suppressed by inhibiting YAP activity

**Fig. 5:**
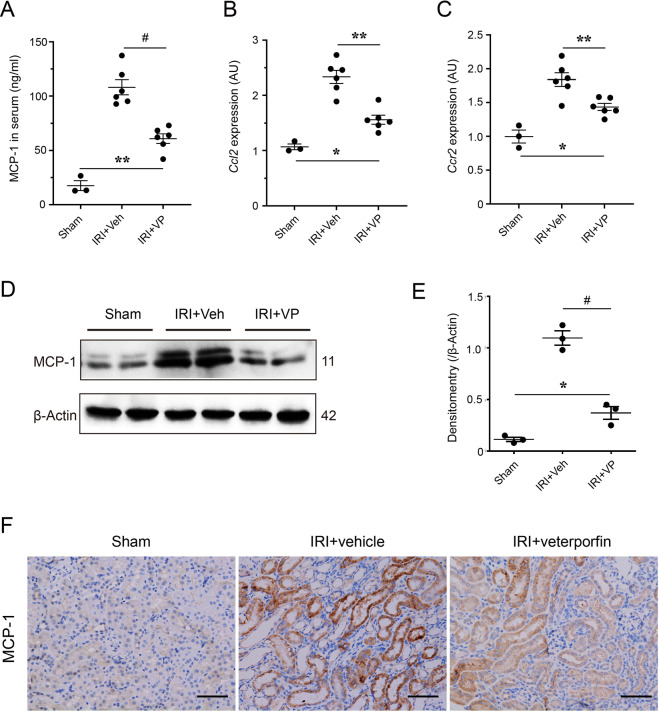
The impact of pharmacological inhibition of YAP on increased renal MCP-1 after AKI. **A** Serum levels of MCP-1 in Sham, IRI + Veh, and IRI + VP mice (*n* = 3 for Sham and *n* = 6, 6 for IRI + Veh and IRI + VP, respectively). **B** Renal mRNA levels of *Ccl2* and **C** *Ccr2* in Sham, IRI + Veh, and IRI + VP mice (*n* = 3 for sham and *n* = 6, 6 for IRI + Veh and IRI + VP, respectively). AU arbitrary units. **D** Expression of renal MCP-1 in Sham, IRI + Veh, and IRI + VP mice. Shown are representative blots from at least three separate experiments with similar results. **E** Quantification and analysis of MCP-1 expression. **F** Representative images of immunohistochemistry staining for renal MCP-1 in Sham, IRI + Veh, and IRI + VP mice. Scale bar = 50 µm in all images of **F**. All values are means ± SEM. *p < 0.05, **p < 0.01, and ^#^*p* < 0.001 defined as significant.

The relationship between YAP activation and MCP-1 overexpression was still not clear. Hence, we also examined the MCP-1 levels in post-AKI mice with YAP pharmacological inhibition. As shown in Fig. 5A, the increased level of serum MCP-1 in AKI mice were declined significantly when the mice were treated by VP (Fig. 5A). In addition, the mRNA levels of *Ccl2* and *Ccr2* in kidneys from VP-treated mice were obviously lower than those from vehicle-treated mice (Fig. 5B, C). Similar to mRNA changes, levels of renal MCP-1 protein were decreased in postischemic kidneys from VP-treated mice compared with the vehicle group (Fig. 5D, E). Moreover, immunohistochemistry staining for MCP-1 showed a significant reduction of tubular MCP-1 expression in AKI mice with VP treatment, compared with the vehicle group (Fig. 5F). These results suggested that in vivo YAP inhibition could suppress tubular MCP-1 increase caused by AKI.

### Oxygen-glucose deprivation and reoxygenation induced YAP activation and MCP-1 increase in vitro

**Fig. 6:**
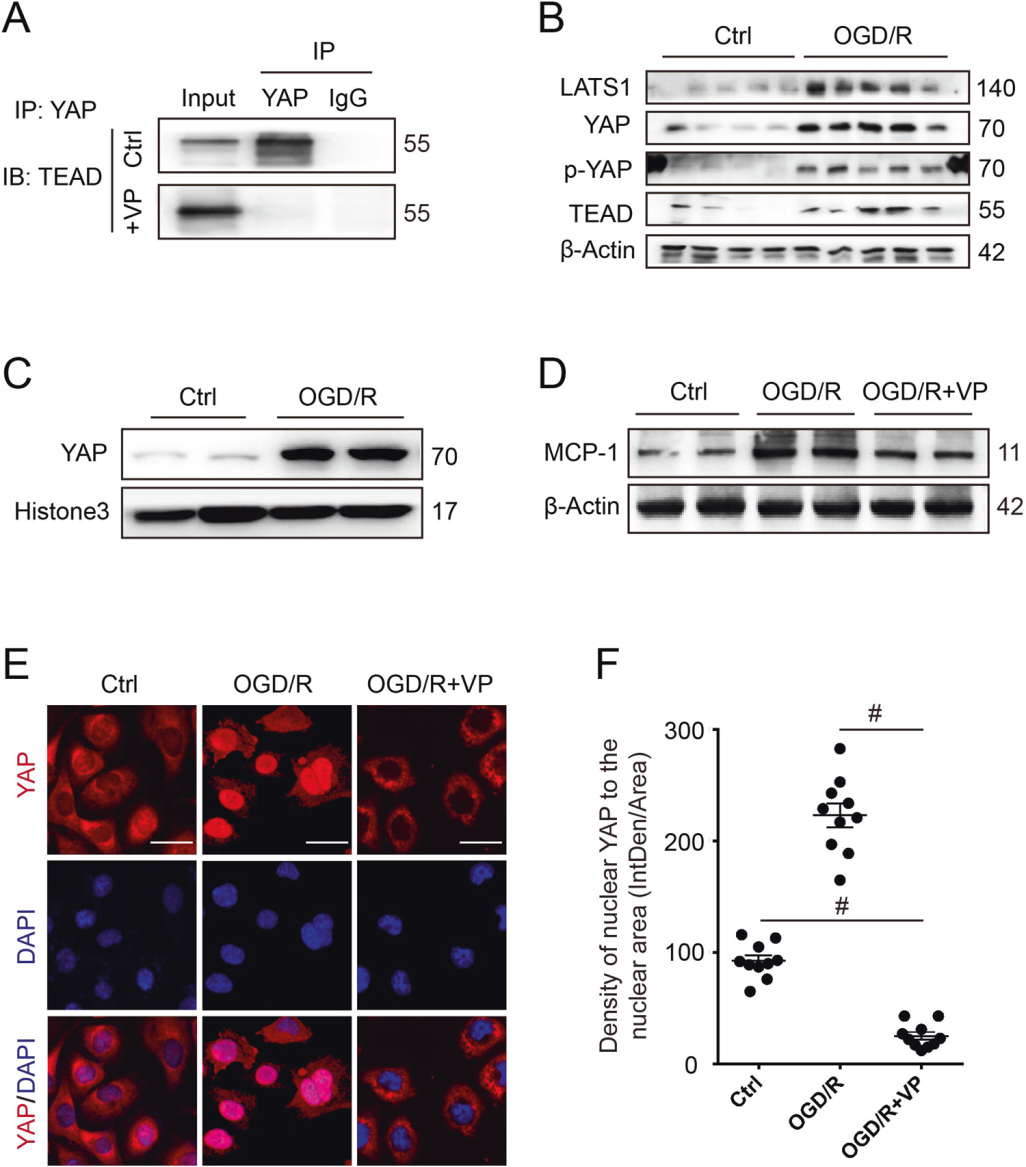
The impact of oxygen-glucose deprivation and reoxygenation (OGD/R) injury on Hippo-YAP pathway and MCP-1 in vitro. **A** The effect of verteporfin (10 µM) on binding interaction between YAP and TEAD in vitro. **B** Cellular expressions of Hippo core proteins (LATS1, YAP, p-YAP[ser127], and TEAD) in HK-2 cells treated with oxygen-glucose deprivation and reoxygenation (OGD/R) injury. **C** Nuclear expression of YAP in HK2 cells after OGD/R treatment. **D** The effect of verteporfin on MCP-1 expression in HK-2 cells after OGD/R. Shown are representative blots from at least three separate experiments with similar results. **E** Representative images of immunofluorescent staining for YAP in verteporfin treated HK-2 cells after OGD/R. **F** Semi-quantification of nuclear YAP expression in immunofluorescent staining (*n* = 10 randomly captured images per group). Scale bar = 20 µm in all images of **E**. All values are means ± SEM. ^#^*p* < 0.001 defined as significant.

In vitro, to mimic IRI, cultured HK-2 cells were exposed to oxygen-glucose deprivation and reoxygenation (OGD/R) stimulation. Firstly, the co-IP analysis revealed that 10 µM VP could effectively block the binding between YAP and TEAD in vitro (Fig. 6A). Western blot showed the protein expression of LATS1, YAP, p-YAP(ser127), and TEAD in HK-2 was increased remarkably, which indicated activation of Hippo pathway after OGD/R stimulation (Fig. 6B). Similar results were also found by using renal TEC from mouse (Supplementary Fig. S2A). In response to OGD/R stress, nuclear YAP distribution was increased, suggesting YAP translocation from cytoplasm to nucleus (Fig. 6C). In contrast, when HK-2 was treated with VP, cellular YAP was primarily localized in the cytosol with minimal distribution in the nucleus (Fig. 6E, F), suggesting that VP could inhibit OGD/R-induced YAP nuclear translocation. Remarkably, MCP-1 expression was significantly increased when HK-2 cells underwent OGD/R (Fig. 6D). However, VP treatment markedly suppressed OGD/R-induced MCP-1 expression (Fig. 6D). In addition, we also found similar results by using TEC (Supplementary Fig. S2A). Thus, OGD/R stress could activate YAP showing nuclear translocation and MCP-1 upregulation.

### MCP-1 was regulated by YAP activity in vitro

**Fig. 7:**
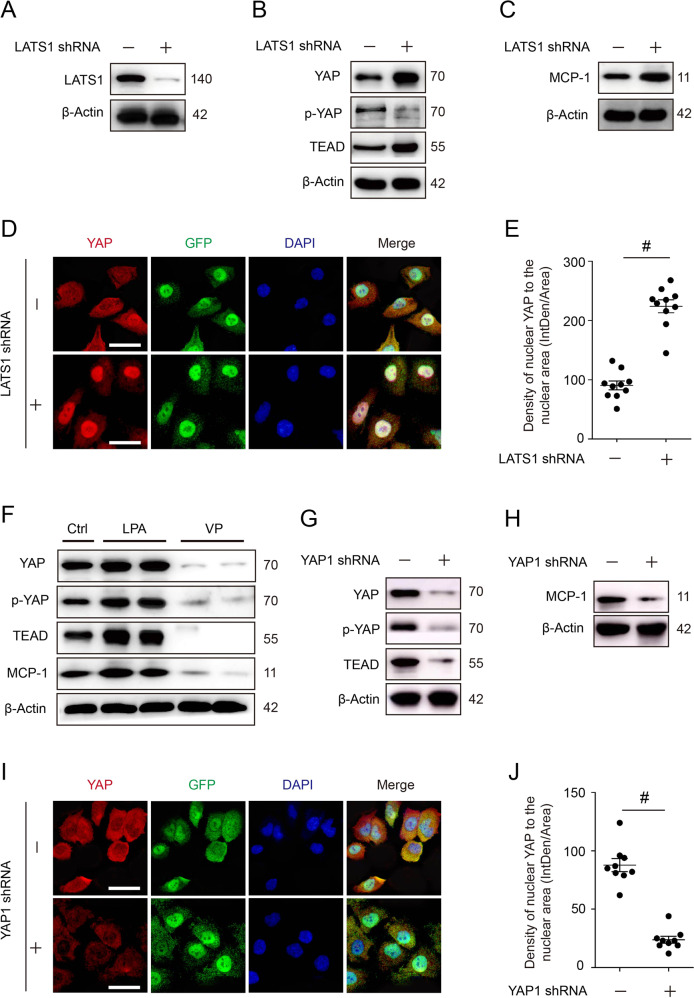
The impact of modulating cellular YAP activity on MCP-1 expression. **A** Cellular expression of LATS1, **B** YAP, p-YAP(ser127), TEAD, and **C** MCP-1 in HK-2 cells with RNA interference (*Lats1* knockdown). Shown are representative blots from at least three separate experiments with similar results. **D** Representative images of immunofluorescent staining for YAP in HK-2 cells with LATS1 silence. **E** Semi-quantification of nuclear YAP expression in cells with LATS1 silence (*n* = 10 randomly captured images per group). **F** Cellular expression of YAP, p-YAP (ser127), TEAD in HK-2 cells treated with LPA or VP. **G** Cellular expression of YAP, p-YAP (ser127), TEAD, and **H** MCP-1 in HK-2 cells with RNA interference (*Yap* knockdown). Shown are representative blots from at least three separate experiments with similar results. **I** Representative images of immunofluorescence staining for YAP in HK-2 cells with *Yap* knockdown. **J** Semi-quantification of nuclear YAP expression in HK-2 cells after silencing YAP expression (*n* = 10 randomly captured images per group). Scale bar = 20 µm in all images of **D** and **I**. All values are means ± SEM. ^#^*p* < 0.001 defined as significant.

To further determine the role of YAP activation on MCP-1 expression in TECs, in vitro study was performed to manipulate the YAP activation or inactivation by RNA interference or inhibitors. Western analysis showed that expression of YAP, TEAD, and MCP-1 significantly increased by silencing LATS1 (Fig. 7A–C). However, cellular p-YAP (ser127) markedly decreased, indicating its dephosphorylation and activation (Fig. 7B). Consistently, cellular immunofluorescence showed YAP nuclear distribution increased in response to LATS1 silence; whereas cytoplasmic level decreased, indicating YAP activation might promote MCP-1 expression (Fig. 7D, E). In contrast, cellular YAP, TEAD, and MCP-1 were significantly reduced when YAP was knockdown (Fig. 7G, H). Compared with negative control, the knockdown group presented a decrease of YAP in both nucleus and cytosol (Fig. 7I, J). In addition, we used LPA or VP to treat HK-2 cells and murine TEC, and found that the expression of YAP, p-YAP (ser127), TEAD, and MCP-1 was all increased in the LPA-treated group but significantly decreased in the VP-treated group, compared with the vehicle-treated group (Fig. 7F and Supplementary Fig. S2B). Taken together, tubular epithelial MCP-1 expression was modulated by YAP activity.

## Discussion

This study explored the potential relationship between the Hippo-YAP pathway and chronic inflammation in maladaptive renal repair. We found severe ischemic AKI-induced tubular maladaptive repair with macrophages infiltration and tubulointerstitial fibrosis. Hippo-YAP pathway was markedly activated and associated with renal inflammation, which might mediate macrophage infiltration and MCP-1 upregulation following ischemic AKI. Inhibiting YAP nuclear translocation attenuated macrophages infiltration and tubulointerstitial fibrosis. Furthermore, we confirmed YAP activity plays a role in controlling MCP-1 expression in vitro.

Renal maladaptive repair has been implicated in CKD progression [5, 28, 29]. In our results, Although YAP activation mainly occurred in abnormal tubules with maladaptive repair, their causal relationship is still not clear. I/R-induced AKI, TECs are sensitive and vulnerable to hypoxia insult [30, 31]. Hypoxia and reoxygenation can lead to paradoxical reactive oxygen species (ROS) overproduction, which further results in oxidative stress and causes cell death [32, 33]. Considering YAP as a regulator controlling cell proliferation, regeneration, and death, we hypothesize that YAP activation in TECs probably strive to resist ROS-induced cell damage and death. A study reported hypoxia could facilitate LATS2 ubiquitination and degradation, which further cause YAP activation [34]. In vitro, we also confirmed that hypoxia and reoxygenation in OGD/R induced Hippo pathway alteration and YAP activation. In addition, YAP overexpression was reported to protect cardiomyocytes against oxidative-induced cell death in murine myocardial ischemic injury [35]. These findings implied that hypoxia and oxidative stress might be explanations for why YAP activation occurred in abnormal tubules with a maladaptive repair.

In addition, Liu et al. reported that hypoxia-induced renal epithelial cell cycle arrest in G2/M in vitro [36]. Recently, G2/M cell cycle arrest is considered an important player in maladaptive tubular repair after AKI [5, 10, 37, 38]. Since YAP activation is also associated with cell growth and proliferation, the question arises whether G2/M arrest induced YAP activation. It is reported that YAP nuclear exclusion or downregulation caused cell cycle arrest in G2/M and increased apoptosis, which suggests that YAP inactivation involves in G2/M arrest [39]. Hence, maladaptive tubular repair with G2/M arrest probably exhibited YAP inactivation. However, our results showed tubular maladaptive repair was associated with YAP activation, which was inconsistent with this suppose. We thought there might be unknown feedback against G2/M arrest and promote its transition through triggering YAP activation. Nevertheless, in spite of these studies, the explanation why YAP is persistently activated associated with tubular maladaptive repair remains to be studied.

The relationship between tubular YAP activation and renal inflammation has not been fully elucidated. We first showed that, in progressive AKI, interstitial macrophages infiltration was correlated with YAP-activated tubules and MCP-1 was markedly upregulated following AKI. More importantly, we found that pharmacological inhibition of YAP in vivo could reduce renal MCP-1 expression and macrophages accumulation. As reported, MCP-1 signaling plays an important role in the crosstalk between injured tubular cells and infiltrating immune cells, which maintains inflammation following AKI [40]. In contrast, blocking MCP-1 function in the AKI model ameliorated the renal pathological changes [40–43]. A previous study demonstrated the genetic deletion of *Mst1/2* led to MCP-1 upregulation and massive macrophages infiltration [20]. In this respect, we hypothesized MCP-1 may mediate YAP activation affecting macrophage’s infiltration. Interestingly, we provided direct in vivo and in vitro evidence that YAP activation could promote MCP-1 production while inhibition or knockdown of YAP, in turn, reduced MCP-1 expression. We further used LPA in vitro to evaluate MCP-1 expression. LPA, known as a Hippo pathway regulator, has an effect to activate YAP by dephosphorylated the LATS1 [44, 45]. The results also strongly indicated that MCP-1 expression was modulated by YAP activity.

Following AKI, YAP probably performs beneficial and detrimental functions on different prognostic stages. It has been demonstrated that YAP plays an essential role in mediating epithelial cell regeneration during kidney recovery from AKI [21, 46]. Despite YAP’s protective effect on the acute phase in AKI, our results exhibited its prominent pro-inflammatory effect on the chronic phase. In the current literature, the timing for the evaluation of renal recovery varies considerably. Some studies determine “recovery” after 3–7 days to make the distinction between transient and persistent AKI [47]. However, there is no clear time point to distinguish the acute recovery phase and the chronic progression phase. Injured tubular cells are not absolutely isochronous, some in proliferation or recovery while some in maladaptive repair or pro-inflammatory stage [48]. It suggests inhibiting YAP in the acute phase too early may have an adverse effect. Indeed, Chen et al. demonstrated that inhibition of YAP function on the first day of IRI by VP delayed renal functional and structural recovery from AKI [21]. We found renal function was partially restored on the 3rd day after IRI, while interstitial inflammatory infiltration and fibrosis was starting to increase (Supplementary Fig. S1). Given that, in order to avoid the adverse impact of YAP inhibition on tubular recovery and to interrupt renal fibrosing as soon as possible, we tested to start VP administration with a low dose at the end of the 3rd day (72 h) after surgery. Indeed, the appropriate usage of VP in mice is still controversial [49, 50]. Eventually, we identified that using VP from the 3rd day effectively attenuated renal inflammatory infiltration and fibrosis, but whether it is the most appropriate time point needs further study.

**Fig. 8:**
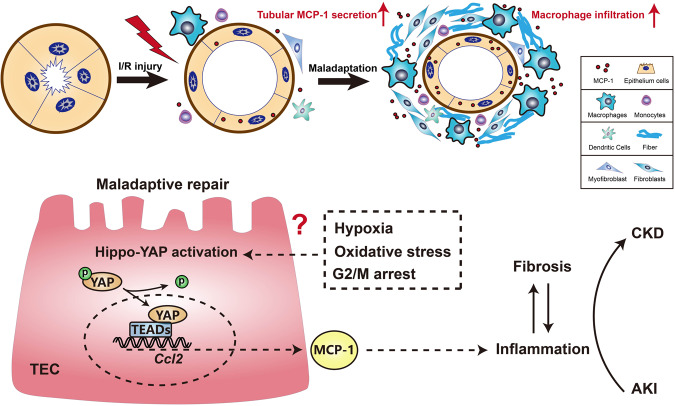
Schematic diagram of YAP-MCP-1 mediating the crosstalk between tubular maladaptation and interstitial infiltration. Following ischemic AKI, YAP activation in tubular epithelial cells causes MCP-1 overexpression and promotes renal macrophage inflammation, which is a driven force for progressive fibrosis and AKI-CKD transition. In addition, hypoxia, oxidative stress, and cell cycle G2/M arrest might be the keys to activate YAP persistently in maladaptive-repaired tubular epithelial cells.

Collectively, we report a link between the tubular Hippo-YAP activation and MCP-1 overproduction, broadening the current knowledge about the crosstalk between maladaptive tubular repair and inflammatory infiltration (Fig. 8). In addition, our studies implicate a low dose of VP as a potential strategy to treat clinic AKI patients at risk for developing CKD. Nevertheless, the mechanism of YAP persistent activation in maladaptive-repaired tubular epitheliums and how to appropriately intervene in YAP overactivation still remain unclear and need to be further explored.

## Supplementary information

Supplementary Information

## References

[1] Bucaloiu ID, Kirchner HL, Norfolk ER, Hartle JE, Perkins RM (2012). Increased risk of death and de novo chronic kidney disease following reversible acute kidney injury. Kidney Int.

[2] Yes HsuCY (2012). AKI truly leads to CKD. J Am Soc Nephrol..

[3] Basile DP, Bonventre JV, Mehta R, Nangaku M, Unwin R, Rosner MH (2016). Progression after AKI: understanding maladaptive repair processes to predict and identify therapeutic treatments. J Am Soc Nephrol..

[4] Venkatachalam MA, Weinberg JM, Kriz W, Bidani AK (2015). Failed tubule recovery, AKI-CKD transition, and kidney disease progression. J Am Soc Nephrol..

[5] Ferenbach DA, Bonventre JV (2015). Mechanisms of maladaptive repair after AKI leading to accelerated kidney ageing and CKD. Nat Rev Nephrol..

[6] Yu SM, Bonventre JV (2020). Acute kidney injury and maladaptive tubular repair leading to renal fibrosis. Curr Opin Nephrol Hypertens..

[7] Sato Y, Yanagita M (2018). Immune cells and inflammation in AKI to CKD progression. Am J Physiol Ren Physiol..

[8] Rabb H, Griffin MD, McKay DB, Swaminathan S, Pickkers P, Rosner MH (2016). Inflammation in AKI: current understanding, key questions, and knowledge gaps. J Am Soc Nephrol..

[9] Huen SC, Cantley LG (2017). Macrophages in renal injury and repair. Annu Rev Physiol..

[10] Liu BC, Tang TT, Lv LL, Lan HY (2018). Renal tubule injury: a driving force toward chronic kidney disease. Kidney Int.

[11] Zhao B, Tumaneng K, Guan KL (2011). The Hippo pathway in organ size control, tissue regeneration and stem cell self-renewal. Nat Cell Biol..

[12] Yu FX, Zhao B, Guan KL (2015). Hippo pathway in organ size control, tissue homeostasis, and cancer. Cell.

[13] Yu FX, Guan KL (2013). The Hippo pathway: regulators and regulations. Genes Dev..

[14] Li L, Zhou J, Li Q, Xu J, Qi J, Bian H (2018). The inhibition of Hippo/Yap signaling pathway is required for magnesium isoglycyrrhizinate to ameliorate hepatic stellate cell inflammation and activation. Biomed. Pharmacother..

[15] Ramjee V, Li D, Manderfield LJ, Liu F, Engleka KA, Aghajanian H (2017). Epicardial YAP/TAZ orchestrate an immunosuppressive response following myocardial infarction. J Clin Invest.

[16] Moroishi T, Hayashi T, Pan WW, Fujita Y, Holt MV, Qin J (2016). The Hippo pathway kinases LATS1/2 suppress cancer immunity. Cell.

[17] Hagenbeek TJ, Webster JD, Kljavin NM, Chang MT, Pham T, Lee H-J (2018). The Hippo pathway effector TAZ induces TEAD-dependentliver inflammation and tumors.. Sci Signal.

[18] Mooring M, Fowl BH, Lum SZC, Liu Y, Yao K, Softic S (2019). Hepatocyte stress increases expression of Yes-associated protein and transcriptional coactivator with PDZ-binding motif in hepatocytes to promote parenchymal inflammation and fibrosis. Hepatology.

[19] Song K, Kwon H, Han C, Chen W, Zhang J, Ma W (2020). Yes-associated protein in Kupffer cells enhances the production of proinflammatory cytokines and promotes the development of nonalcoholic steatohepatitis. Hepatology.

[20] Kim W, Khan SK, Liu Y, Xu R, Park O, He Y (2018). Hepatic Hippo signaling inhibits protumoural microenvironment to suppress hepatocellular carcinoma. Gut.

[21] Chen J, You H, Li Y, Xu Y, He Q, Harris RC (2018). EGF receptor-dependent YAP activation is important for renal recovery from AKI. J Am Soc Nephrol..

[22] Zheng Z, Deng G, Qi C, Xu Y, Liu X, Zhao Z (2019). Porous Se@SiO2 nanospheres attenuate ischemia/reperfusion (I/R)-induced acute kidney injury (AKI) and inflammation by antioxidative stress. Int J Nanomed..

[23] Choi IY, Yan H, Park YK, Kim WK (2009). Sauchinone reduces oxygen-glucose deprivation-evoked neuronal cell death via suppression of intracellular radical production. Arch Pharm Res.

[24] Lin M, Ling J, Geng X, Zhang J, Du J, Chen L (2019). RTN1-C is involved in high glucose-aggravated neuronal cell subjected to oxygen-glucose deprivation and reoxygenation injury via endoplasmic reticulum stress. Brain Res Bull..

[25] Markó L, Vigolo E, Hinze C, Park JK, Roël G, Balogh A (2016). Tubular epithelial NF-κB activity regulates ischemic AKI. J Am Soc Nephrol..

[26] Kong W, Haschler TN, Nürnberg B, Krämer S, Gollasch M, Markó L (2019). Renal fibrosis, immune cell infiltration and changes of TRPC channel expression after unilateral ureteral obstruction in Trpc6-/- mice. Cell Physiol Biochem.

[27] Liu J, Gao M, Nipper M, Deng J, Sharkey FE (2019). Activation of the intrinsic fibroinflammatory program in adult pancreatic acinar cells triggered by Hippo signaling disruption. PLoS Biol..

[28] Liu J, Kumar S, Dolzhenko E, Alvarado GF, Guo J, Lu C, et al. Molecular characterization of the transition from acute to chronic kidney injury following ischemia/reperfusion. JCI Insight. 2017;2:e94716. 10.1172/jci.insight.94716 PMC5612583 28931758

[29] Sato Y, Takahashi M, Yanagita M (2020). Pathophysiology of AKI to CKD progression. Semin Nephrol..

[30] Devarajan P (2006). Update on mechanisms of ischemic acute kidney injury. J Am Soc Nephrol..

[31] Yamamoto K, Tomita N, Yoshimura S, Nakagami H, Taniyama Y, Yamasaki K (2004). Hypoxia-induced renal epithelial cell death through caspase-dependent pathway: role of Bcl-2, Bcl-xL and Bax in tubular injury. Int J Mol Med.

[32] Yoshitomi T, Hirayama A, Nagasaki Y (2011). The ROS scavenging and renal protective effects of pH-responsive nitroxide radical-containing nanoparticles. Biomaterials.

[33] Gu Y, Huang F, Wang Y, Chen C, Wu S, Zhou S (2018). Connexin32 plays a crucial role in ROS-mediated endoplasmic reticulum stress apoptosis signaling pathway in ischemia reperfusion-induced acute kidney injury. J Transl Med.

[34] Ma B, Cheng H, Gao R, Mu C, Chen L, Wu S (2016). Zyxin-Siah2-Lats2 axis mediates cooperation between Hippo and TGF-β signalling pathways. Nat Commun..

[35] Del ReDP, Yang Y, Nakano N, Cho J, Zhai P, Yamamoto T (2013). Yes-associated protein isoform 1 (Yap1) promotes cardiomyocyte survival and growth to protect against myocardial ischemic injury. J Biol Chem..

[36] Liu T, Liu L, Liu M, Du R, Dang Y, Bai M (2019). MicroRNA-493 targets STMN-1 and promotes hypoxia-induced epithelial cell cycle arrest in G(2)/M and renal fibrosis. FASEB J..

[37] Yang L, Besschetnova TY, Brooks CR, Shah JV, Bonventre JV (2010). Epithelial cell cycle arrest in G2/M mediates kidney fibrosis after injury. Nat Med.

[38] Moonen L, D’Haese PC, Vervaet BA. Epithelial cell cycle behaviour in the injured kidney. Int J Mol Sci. 2018;19:2038. 10.3390/ijms19072038 PMC6073451 30011818

[39] Tang X, Sun Y, Wan G, Sun J, Sun J, Pan C (2019). Knockdown of YAP inhibits growth in Hep-2 laryngeal cancer cells via epithelial-mesenchymal transition and the Wnt/β-catenin pathway. BMC Cancer.

[40] Xu L, Sharkey D, Cantley LG (2019). Tubular GM-CSF promotes late MCP-1/CCR2-mediated fibrosis and inflammation after ischemia/reperfusion injury. J Am Soc Nephrol..

[41] Haller H, Bertram A, Nadrowitz F, Menne J (2016). Monocyte chemoattractant protein-1 and the kidney. Curr Opin Nephrol Hypertens..

[42] Deshmane SL, Kremlev S, Amini S, Sawaya BE (2009). Monocyte chemoattractant protein-1 (MCP-1): an overview. J Interferon Cytokine Res.

[43] Furuichi K, Wada T, Iwata Y, Kitagawa K, Kobayashi K, Hashimoto H (2003). CCR2 signaling contributes to ischemia-reperfusion injury in kidney. J Am Soc Nephrol..

[44] Cai H, Xu Y (2013). The role of LPA and YAP signaling in long-term migration of human ovarian cancer cells. Cell Commun Signal.

[45] Hwang SM, Jin M, Shin YH, Ki Choi S, Namkoong E, Kim M (2014). Role of LPA and the Hippo pathway on apoptosis in salivary gland epithelial cells. Exp Mol Med.

[46] Xu J, Li PX, Wu J, Gao YJ, Yin MX, Lin Y (2016). Involvement of the Hippo pathway in regeneration and fibrogenesis after ischaemic acute kidney injury: YAP is the key effector. Clin Sci..

[47] Forni LG, Darmon M, Ostermann M (2017). Oudemans-van Straaten HM, Pettilä V, Prowle JR, et al. Renal recovery after acute kidney injury. Intensive Care Med.

[48] Polichnowski AJ, Lan R, Geng H, Griffin KA, Venkatachalam MA, Bidani AK (2014). Severe renal mass reduction impairs recovery and promotes fibrosis after AKI. J Am Soc Nephrol..

[49] Liu-Chittenden Y, Huang B, Shim JS, Chen Q, Lee SJ, Anders RA (2012). Genetic and pharmacological disruption of the TEAD-YAP complex suppresses the oncogenic activity of YAP. Genes Dev..

[50] Morishita T, Hayakawa F, Sugimoto K, Iwase M, Yamamoto H, Hirano D (2016). The photosensitizer verteporfin has light-independent anti-leukemic activity for Ph-positive acute lymphoblastic leukemia and synergistically works with dasatinib. Oncotarget.

