# Supplementary material for: Hippo-YAP/MCP-1 mediated tubular maladaptive repair promote inflammation in renal failed recovery after ischemic AKI
Source: Cell Death Dis. 2021 Jul 30;12(8):754. doi: 10.1038/s41419-021-04041-8 (PMC8324794; doi:10.1038/s41419-021-04041-8)
Supplement: Supplementary file 1 — Supplementary Information [file 41419_2021_4041_MOESM1_ESM.docx]

Zheng et al.

**Supplementary Information**

**Supplementary Figure S1.** Evaluation of renal function, histopathology, inflammatory infiltration, and fibrosis in early 3 days after IRI. (A) Serum levels of creatinine and (B) Blood urea nitrogen after IRI. (C) Representative images of HE, F4/80, and α-SMA staining in injured kidneys. Scale bar=50 µm in all images of C. All values are means ± SEM. **p*＜0.05, ***p*＜0.01 and #*p*＜0.001 defined as significant.

**Supplementary Figure S1**

**
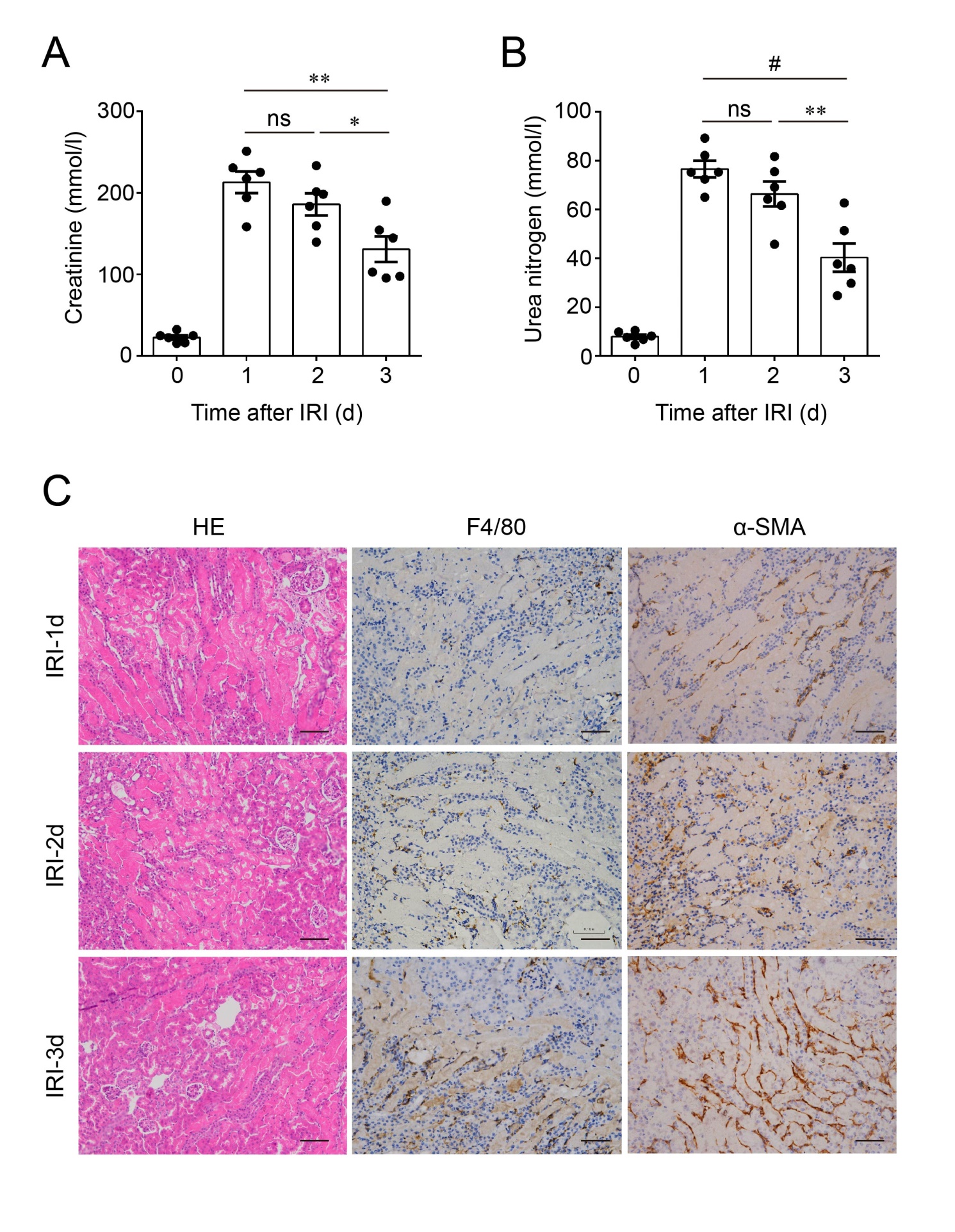
**

Zheng et al.

**Supplementary Figure S2.** The response of Hippo pathway or MCP-1 in murine TEC to OGD/R or YAP manipulation by agonists or inhibitors. (A) Western blot examined the change of Hippo pathway or MCP-1 in murine TEC with OGD/R treatment. (B) Western blot examined the change of Hippo pathway or MCP-1 in murine TEC treated by LPA or VP. All values are means ± SEM. **p*＜0.05, ***p*＜0.01 and #*p*＜0.001 defined as significant *versus* control group.

**Supplementary Figure S2**

**
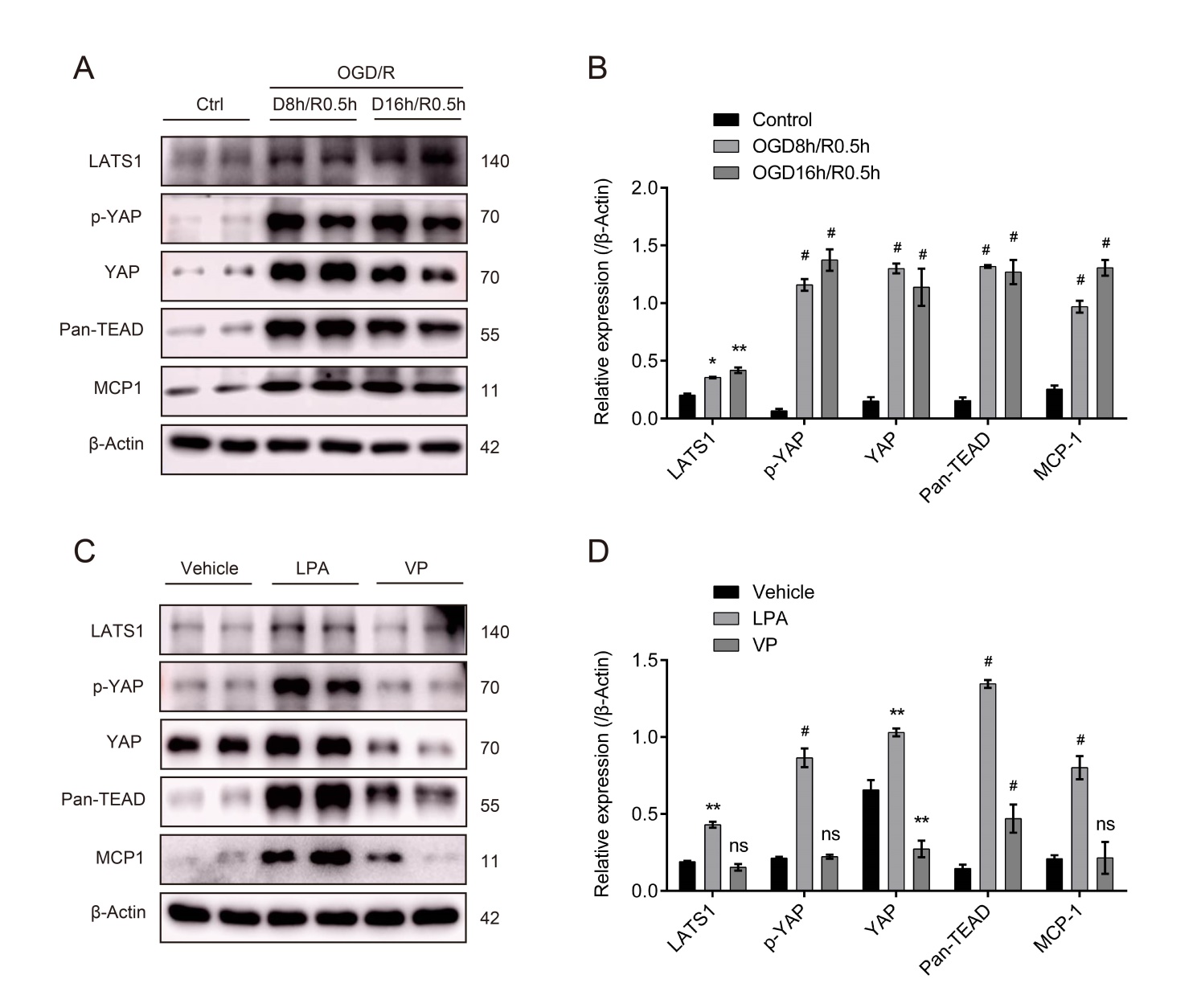
**

Zheng et al.

**Supplementary Table S1.** The primer sequence of murine genes (*Yap1, Ccl2, Ccr2, β-Act*).

**Supplementary Table S1**

**
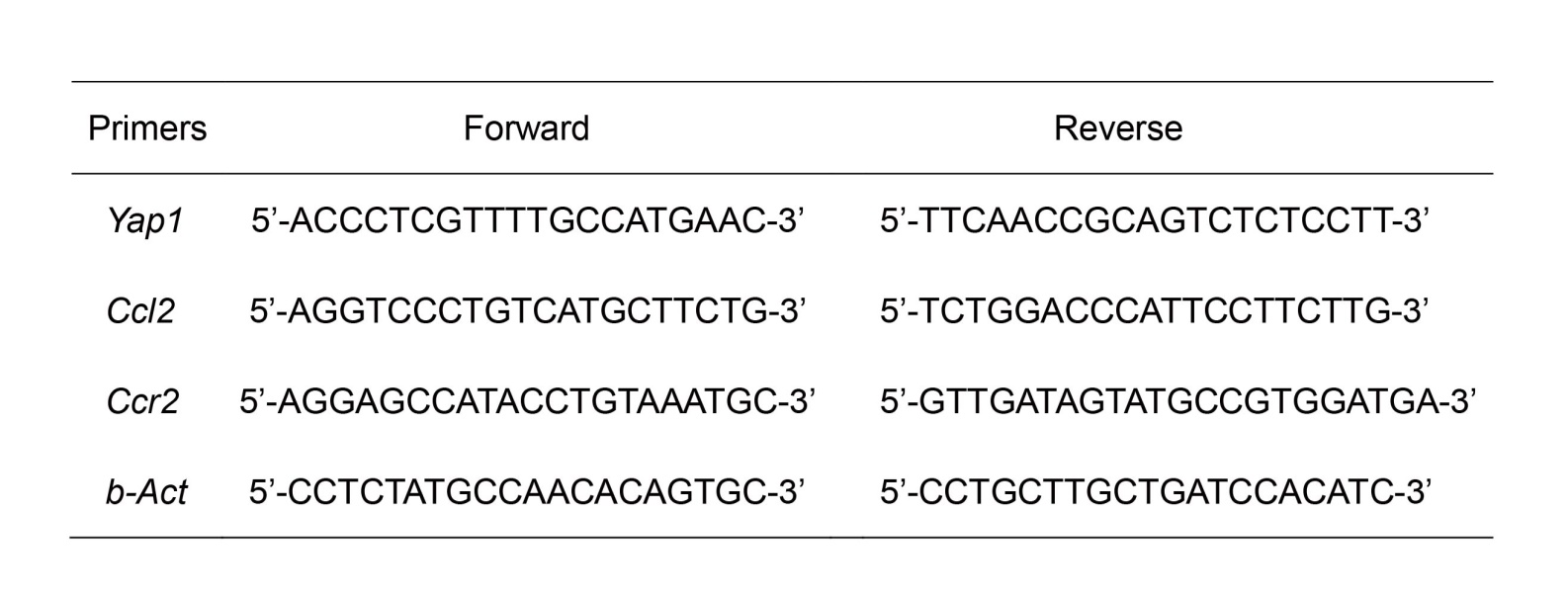
**
